# Acute Infectious Gastroenteritis Potentiates a Crohn's Disease Pathobiont to Fuel Ongoing Inflammation in the Post-Infectious Period

**DOI:** 10.1371/journal.ppat.1005907

**Published:** 2016-10-06

**Authors:** Cherrie L. Small, Lydia Xing, Joseph B. McPhee, Hong T. Law, Brian K. Coombes

**Affiliations:** 1 Michael G. DeGroote Institute for Infectious Disease Research, Hamilton, Ontario, Canada; 2 Department of Biochemistry and Biomedical Sciences, McMaster University, Hamilton, Ontario, Canada; 3 Farncombe Family Digestive Health Research Institute, Hamilton, Ontario, Canada; University of California Davis School of Medicine, UNITED STATES

## Abstract

Crohn’s disease (CD) is a chronic inflammatory condition of diverse etiology. Exposure to foodborne pathogens causing acute gastroenteritis produces a long-term risk of CD well into the post-infectious period but the mechanistic basis for this ongoing relationship to disease onset is unknown. We developed two novel models to study the comorbidity of acute gastroenteritis caused by *Salmonella* Typhimurium or *Citrobacter rodentium* in mice colonized with adherent-invasive *Escherichia coli* (AIEC), a bacterial pathobiont linked to CD. Here, we show that disease activity in the post-infectious period after gastroenteritis is driven by the tissue-associated expansion of the resident AIEC pathobiont, with an attendant increase in immunopathology, barrier defects, and delays in mucosal restitution following pathogen clearance. These features required AIEC resistance to host defense peptides and a fulminant inflammatory response to the enteric pathogen. Our results suggest that individuals colonized by AIEC at the time of acute infectious gastroenteritis may be at greater risk for CD onset. Importantly, our data identify AIEC as a tractable disease modifier, a finding that could be exploited in the development of therapeutic interventions following infectious gastroenteritis in at-risk individuals.

## Author Summary

Western societies have a disproportionately high rate of inflammatory bowel disease (IBD), with growing incidence especially in the adolescent population. A large body of evidence supports the view that bacteria in the gut participate in the pathophysiology of human bowel diseases. The unifying concept is chronic inflammation that is driven by microbial stimulation of the mucosal immune system. However, the mechanisms by which pathogenic or commensal microbes work in concert with each other and with host responses to perpetuate this inflammation is not well known. Adherent-invasive *E*. *coli* (AIEC) are Crohn’s disease (CD)-associated bacteria that are implicated in disease pathology. AIEC are pro-inflammatory and may play a central role in maintaining chronic inflammation in response to other CD risk factors, such as acute infectious gastroenteritis. Here, we show that indeed, acute infectious gastroenteritis creates an inflammatory environment in the gut that drives AIEC expansion and worsens disease severity. The increase in disease severity strictly correlates with this AIEC bloom because blocking this bloom by sensitizing AIEC to host defenses also improves the health status of the host. The long time period between recovery from acute gastroenteritis and new onset CD may allow for targeted interventions to mitigate the risk of CD in AIEC-positive individuals.

## Introduction

Inflammation in Crohn’s disease (CD) can involve the small and large bowel and is accompanied by changes in the microbial composition and distribution at these sites [1–3]. Clinical observations have been consistent in finding increased numbers of bacteria associated with the epithelial mucosa in Crohn’s patients, including members of the *Enterobacteriaceae* that are enriched in virulence and secretion pathways as determined by culture and molecular methods [4–6]. Although a detailed microbiologic analysis of this population has not been done and their role in disease has not been described, these data imply that the environment favoring bacterial expansion at the mucosal surface is inflammatory in nature and that this particular bacterial bloom is of pathogenic significance in the disease.

Among bacteria linked to CD, adherent-invasive *E*. *coli* (AIEC) have emerged as having likely pathogenic significance. Since its discovery in 1998 [7], several laboratories have reported a higher prevalence of AIEC in CD patients compared to healthy subjects and confirmed their pro-inflammatory potential [5, 8, 9]. Although AIEC share evolutionary ancestry with extraintestinal pathogenic *E*. *coli* [10], their infection biology suggests a pathobiont lifestyle that is distinct from frank enteric pathogens. A growing body of work indicates that different host environments can select for AIEC. For example, AIEC isolated from adults [5, 10–12], children [13, 14], and companion animals [15] exhibit a degree of genetic diversity that has made it difficult to discriminate this *E*. *coli* pathotype at a molecular level. AIEC can also be isolated from seemingly healthy individuals (albeit much less frequently than in CD), implying that interactions between AIEC and other microbes or host genes might be needed to elicit their pathogenic character.

CD is more common in individuals exposed to acute infectious gastroenteritis caused by *Salmonella* and other enteric pathogens, sometimes with onset times on the order of years after the infectious episode [16–18]. The mechanistic basis for this long-term risk association following an acute event lasting ~2 weeks is unresolved, however one possibility is that resident gut microbes could perpetuate inflammatory reactions in the post-infectious period. This relates to several clinical observations [19, 20]. First, acute infectious gastroenteritis increases the risk of CD onset, often through an intermediate state of post-infectious-irritable bowel syndrome (IBS) [16–18]; second, gastroenteritis causes inflammation that selectively disrupts the resident intestinal microbiota in favor of some members of the *Enterobacteriaceae* [21, 22]; and third, intestinal infections with frank enteric pathogens have been implicated as a probable cause of relapse in inflammatory bowel disease (IBD) patients [23, 24].

We developed two new models to study comorbidity following acute infectious gastroenteritis in hosts colonized by an AIEC pathobiont from a Crohn’s disease patient. We show that AIEC-colonized mice that develop infectious gastroenteritis in response to either *Salmonella enterica* serovar Typhimurium (*S*. Typhimurium), or the attaching and effacing mouse pathogen *Citrobacter rodentium*, have a worsened outcome compared to AIEC-naïve animals exposed to the same infection stimuli. Bacterial gastroenteritis induced an AIEC bloom in the ileum and colon accompanied by an increase in disease severity. The increase in disease severity strictly correlated with this AIEC bloom because blocking AIEC expansion by sensitizing the bacteria to host defenses ameliorated disease status, providing a direct correlation between AIEC and host damage. These data are the first to show that acute gastroenteritis modulates AIEC levels in the colonized gut, and establish AIEC as an active yet tractable disease modifier in response to acute infectious gastroenteritis.

## Results

### AIEC modifies disease outcome following *S*. Typhimurium-induced gastroenteritis

**Fig 1:**
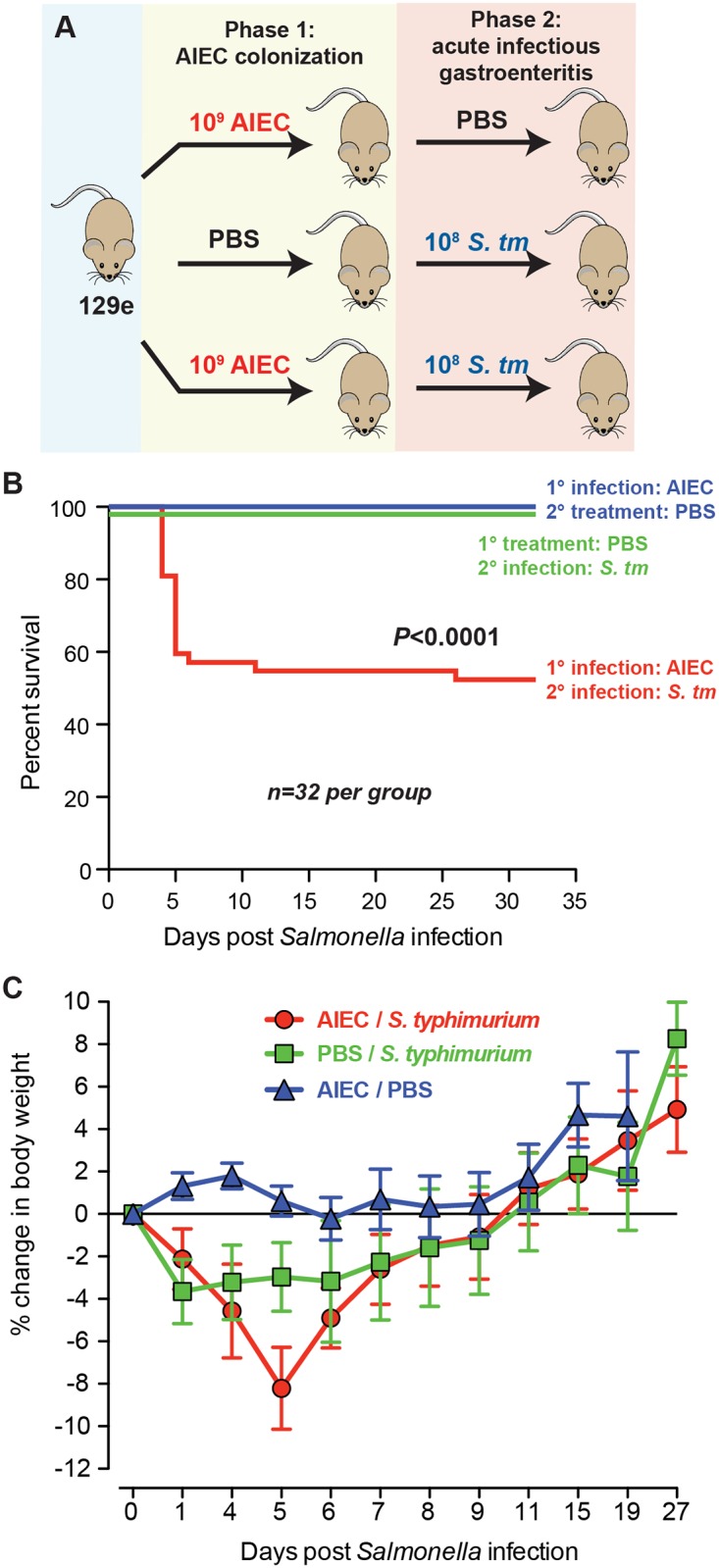
AIEC modifies disease outcome following acute gastroenteritis. (A). Infection scheme: NRAMP+ 129e mice were colonized with 2 x 10^9^ cfu of AIEC strain NRG857c for 14 days to establish chronic colonization and low-grade intestinal inflammation (Phase 1). Control mice remained AIEC-naïve. AIEC-colonized and control mice were exposed to acute infectious gastroenteritis with 0.8 x 10^8^ cfu of *Salmonella enterica* serovar Typhimurium (Phase 2). (B) Kaplan-Meier survival plots of 129e mice after *Salmonella* infection. *n* = 32 mice per group from 6 independent experiments (p<0.0001, log rank). (C) Percent change in body weight was monitored up to 27 days post-*Salmonella* infection. Data is expressed as a mean ± SEM of 5 mice per group from 8 independent experiments.

To study the link between acute infectious gastroenteritis and susceptibility to CD we developed a co-infection model that leveraged a model of chronic colonization of wild type mice with AIEC strain NRG857c [25]. This isolate from a patient with Crohn’s disease colonizes both 129e and C57BL/6 mouse strains, allowing for non-lethal secondary infections with *S*. Typhimurium and *C*. *rodentium*, respectively. First, groups of 129e mice were colonized with AIEC for 14 days or remained AIEC-naïve, and then exposed to *S*. Typhimurium to invoke acute infectious gastroenteritis (Fig 1A). Polymicrobial infection with AIEC and *S*. Typhimurium lead to significantly worsened clinical outcome in 129e mice compared to mono-infected mice. All mice infected with either AIEC or *S*. Typhimurium alone survived for the duration of the experiment (35 days), whereas ~ 50% of the co-infected mice died over a 6-week period after the secondary *Salmonella* infection (Fig 1B). More severe disease was evident in the co-infected cohort, which had diarrhoea, dehydration, hunched posture, and significant body weight loss at ~ day 5 after infectious gastroenteritis was initiated (Fig 1C). Overall these results indicated that prior colonization by AIEC worsened the clinical outcome of infectious gastroenteritis.

### Prior AIEC colonization worsens pathology following *S*. *Typhimurium* infection

**Fig 2:**
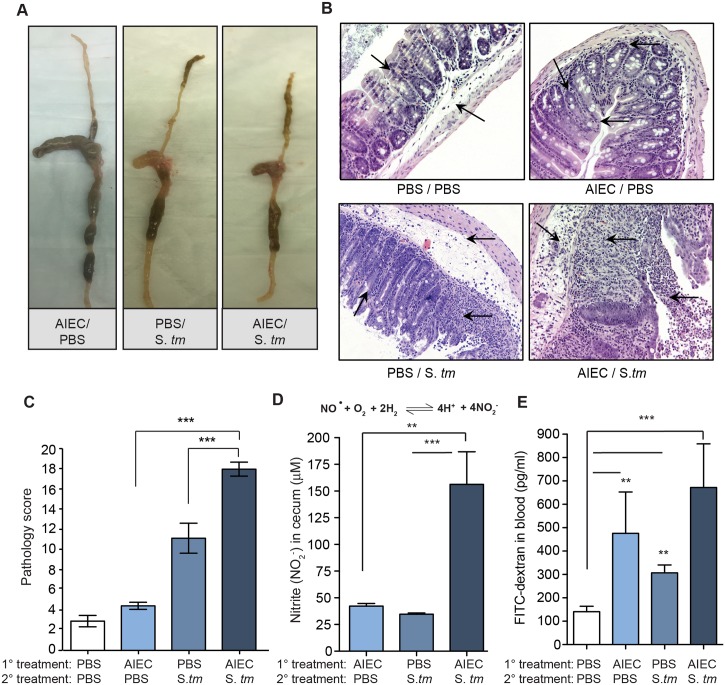
Prior AIEC colonization worsens pathology following *S*. *Typhimurium* infection. (A) Gross pathology in the small and large intestine of 129e mice 5 days after *Salmonella* infection. (B) H&E-stained cecal sections from 4 experimental groups of 129e mice (as indicated). Images are representative of 5 mice per group from 4 independent experiments. Arrows indicate submucosa edema, desquamation, epithelial sloughing, crypt hyperplasia, and inflammatory cellular infiltrates in the lumen and mucosa. Original magnification 40x. (C) Cecal pathology was scored from H&E stained sections taken from day 5. Data are the mean with ± SEM for 5 views per mouse and 5 mice per group. ***p<0.001 (Mann-Whitney). (D) Nitrite concentration in cecal samples on day 5 after *Salmonella* infection. Treatment groups are indicated. Data are the means with SEM. **p<0.01, ***p<0.001 (Mann-Whitney). (E) FITC-dextran concentration in the serum following oral gavage in the indicated groups. Data are the means with SEM. **p<0.01, ***p<0.001 (Mann-Whitney).

Infection of AIEC-colonized mice with *S*. Typhimurium was accompanied by non-segmented watery stool, a reduction in cecal and colon size and a hardened rubbery texture of the gut with occasional ulcerations (Fig 2A). Histochemical analysis performed on H&E-stained cecal sections from *S*. Typhimurium infected mice showed only focal submucosal edema and mild to moderate epithelial hyperplasia, whereas co-infected mice had dramatic epithelial destruction along with extensive transmural inflammation, edema, and loss of crypt architecture (Fig 2B). When quantified, the histopathology in co-infected mice was significantly greater than in mice monocolonized with either AIEC or *S*. Typhimurium (Fig 2C).

Nitric oxide is a biomarker for active bowel inflammation and is directly correlated with disease activity in IBD patients [26]. We measured nitrite levels in the cecum from animals either monocolonized with AIEC or *S*. Typhimurium, or co-infected with both. Cecal nitrite levels were significantly higher in mice co-infected with AIEC and *S*. Typhimurium, consistent with the immunopathology observed previously (Fig 2D). Ulceration and damage to the mucosal epithelium in co-infected mice suggested permeability defects in the epithelial barrier. To test this we gavaged mice with fluorescein isothicyanate (FITC)-dextran (FD4) and measured translocation of FD4 from the gut lumen to the serum. These results showed a significant increase in serum FD4 in all infected mouse groups compared to uninfected control mice, with the largest increase seen in *S*. Typhimurium-infected mice pre-colonized with AIEC (Fig 2E).

### Acute infectious gastroenteritis causes expansion of the tissue-associated AIEC population

**Fig 3:**
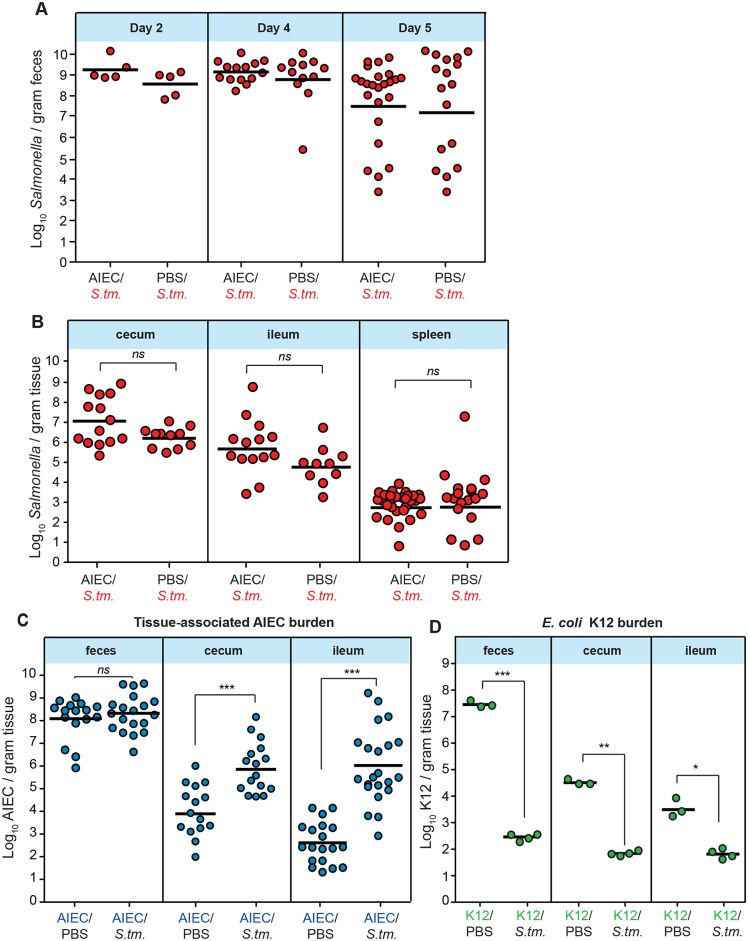
Tissue-associated expansion of AIEC following *Salmonella gastroenteritis*. (A) Fecal *Salmonella* loads on day 2, 4, and 5 after infection. Each data point is from one animal and represents 3–4 independent experiments. (B) Tissue-associated *Salmonella* burdens in the cecum, ileum, and spleen on day 5 after *Salmonella* infection. Each point is from one animal and data represents 3–4 independent experiments. (C) Tissue-associated AIEC burden in feces, cecum and ileum on day 5 after *Salmonella* infection. Each point is from one animal and data represent 3–4 independent experiments. (D) Tissue-associated *E*. *coli* K12 in feces, cecum and ileum on day 5 after *Salmonella* infection. Each point is from one animal. *ns* not significantly different between comparison groups, *p<0.05, **p<0.01, ***p< 0.001 (Mann-Whitney test).

The higher rates of mortality seen in *S*. Typhimurium-infected 129e mice harbouring resident AIEC was reminiscent of mortality profiles in susceptible mouse lines such as C57BL/6 that do not constrain *S*. Typhimurium and develop lethal levels of bacteremia. Therefore, we hypothesized that prior AIEC colonization was driving a *Salmonella*-induced disease process, however this was inconsistent with the *S*. Typhimurium colonization data. By measuring *S*. Typhimurium loads in the feces over time we found that 129e controlled *S*. Typhimurium similarly in both AIEC-colonized and AIEC-naïve animals, with colonization rates similar to that found in other studies [27]. Indeed, *Salmonella* colonization levels decreased over time in line with the ability of 129e mice to restrict the growth of *S*. Typhimurium [28] (Fig 3A). Consistent with these data, the tissue-associated levels of *S*. Typhimurium in the cecum, ileum and spleen were similar in monocolonized and co-infected animals (Fig 3B), indicating that AIEC did not alter the ability of the host to control *S*. Typhimurium.

After AIEC colonization, 129e mice maintain stable AIEC levels [25]. In contrast to *Salmonella* loads in our model, we found dramatic and reproducible increases in the levels of AIEC following acute infectious gastroenteritis that was reflected only in the tissue-associated bacterial population. This bacterial bloom was not evident in the bulk luminal population isolated from the feces, however we found a 2-log and 3-log increase in tissue-associated AIEC levels in the cecum and ileum, respectively (Fig 3C). Culture-independent methods have found that ileal CD lesions are enriched in *Enterobacteriacae* containing pathobiont-associated properties such as adhesion and invasion [5], a finding that has been confirmed in functional metagenomic studies [4]. To test whether this AIEC bloom was specific to this pathobiont or reflected a more generalized permissive niche for *E*. *coli*, we repeated these experiments with mice colonized with a rifampicin-resistant derivative of the human commensal *E*. *coli* K12. Despite showing stable colonization levels before infectious gastroenteritis, *Salmonella* infection caused a dramatic decrease in *E*. *coli* K12 levels in both the feces and tissue-associated samples (Fig 3D), which was in contrast to the bloom seen by AIEC. These results are consistent with the susceptibility of commensal *E*. *coli* to *Salmonella*-induced inflammation, leading to *E*. *coli* killing in the inflamed gut [29]. Together these results support the hypothesis that acute infectious gastroenteritis creates a specialized niche permissive for the AIEC pathobiont with enhanced fitness under inflamed conditions, and are consistent with the selective increase in invasive tissue-associated *E*. *coli* in CD biopsies [4, 5].

### Prior AIEC colonization leads to heightened cellular and proinflammatory cytokine responses in the cecum following *S*. *Typhimurium* infection

**Fig 4:**
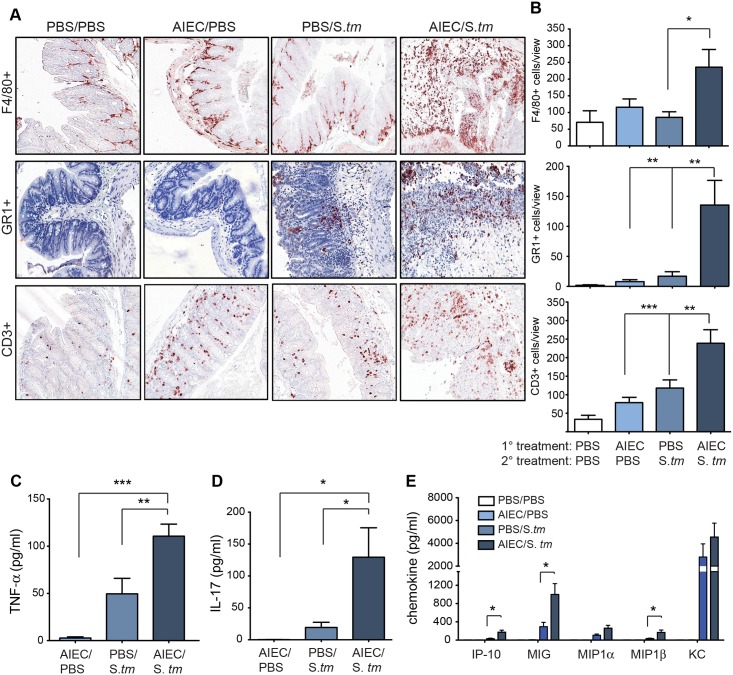
Prior AIEC colonization leads to heightened cellular and proinflammatory cytokine responses. (A) Immunohistochemical staining of cecal tissue for F4/80+, GR1+, and CD3+ cells. Each image is representative of 2 independent experiments with 5 mice per group. Original magnification 200x. (B) Quantification of immunohistochemical staining. Data is from 6–8 views per section from 5 mice. *p<0.05, **p<0.01, ***p<0.001(Mann-Whitney test). (C) TNF-α, (D) IL-17 and (E) chemokines were measured using ELISA from supernatants after overnight incubation of explanted ceca. Data are the means ± SEM of 5 mice per group from 3 separate experiments. *p<0.05, **p<0.01, ***p<0.001 (one way ANOVA with Tukey for comparisons between groups).

CD is characterized by a heightened inflammatory state in the gut [30]. We next characterized the inflammatory environment in the gut that selected for AIEC outgrowth five days after infectious gastroenteritis was initiated. Immunohistochemical analysis showed a significant increase in the number of infiltrated F4/80^+^, GR1^+^, and CD3^+^ cells in the cecum of AIEC-colonized mice at day 5 after *S*. Typhimurium infection, compared to the monocolonized or uninfected control groups (Fig 4A and 4B). These immune cell populations were heavily concentrated in the lamina propria compared to the singly infected groups as well as the PBS control group. The lamina propria of CD lesions contains elevated levels of tumour necrosis factor-α (TNF-α) and IL-17, which might be involved in the maintenance of transmural intestinal inflammation [27, 31]. There was a significant increase in TNF-α and IL-17 in cecal supernatants from AIEC-colonized mice exposed to *Salmonella* gastroenteritis compared to either monocolonized mice or uninfected controls (Fig 4C and 4D). TNF-α is a proinflammatory cytokine involved in the secondary induction of chemokines and is the target of biologic therapy used in CD [32]. Consistent with the TNF-α and cellular responses seen in the cecum there was increased levels of released MIG, IP-10 and MIP-1β chemokines in AIEC colonized mice exposed to *S*. Typhimurium (Fig 4E). Together these data indicated that prior AIEC status of the host had a dramatic impact on disease outcome following acute infectious gastroenteritis. Whereas AIEC-naïve hosts developed a self-limiting infectious gastroenteritis, hosts with indwelling AIEC had heightened immunopathology, immune cell infiltration, and a marked expansion of tissue-associated AIEC loads in the gut.

### Resistance to host defense peptides is required for AIEC expansion in the inflamed gut and immunopathology

**Fig 5:**
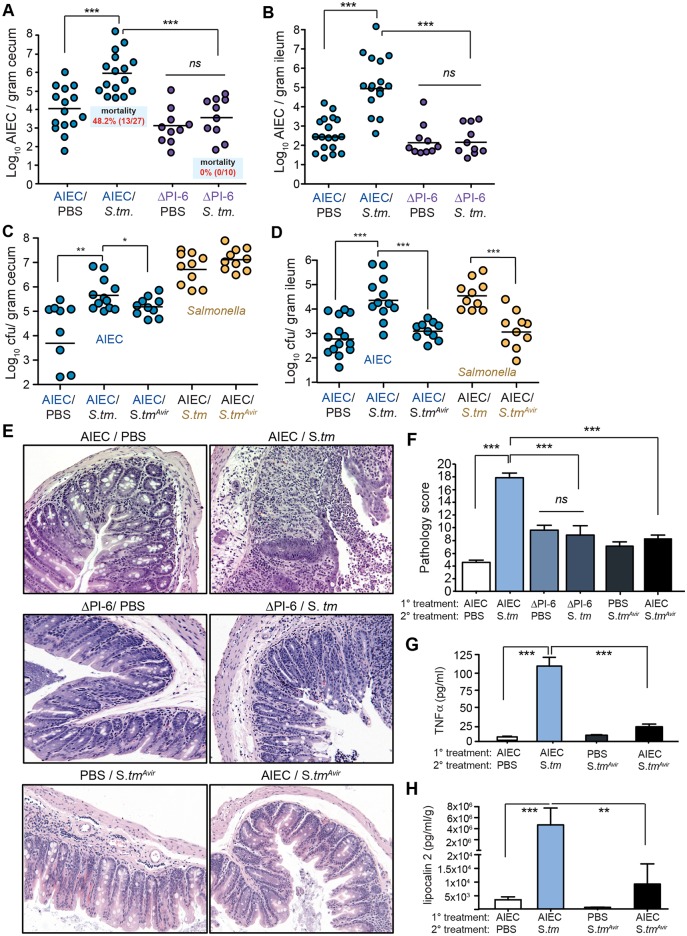
Resistance to host defense peptides is required for AIEC expansion in the inflamed gut and immunopathology. 129e mice were colonized with wild type AIEC or the peptide-sensitive ΔPI-6 mutant and then infected with *Salmonella*. Survival was assessed over 35 days and the tissue burden of AIEC was determined in the cecum (A) and the ileum (B). Each point represents one animal and the data represents 3 independent experiments. ***p< 0.001 (Mann-Whitney test). 129e mice were colonized with AIEC and then infected with either wild type *Salmonella* or a mutant that is less proinflammatory, *S*.*tm* ^*avir*^. Tissue burden of AIEC (blue circles) and *Salmonella* (yellow circles) was determined in the cecum (C) and the ileum (D). Each point represents one animal and the data represents 2 independent experiments. *p<0.05; **p<0.01; ***p< 0.001 (Mann-Whitney test). (E) H&E-stained tissue samples from cecal tips are shown for the indicated infection groups. Original magnification 200x. (F) Quantification of histopathology from part E. Data are the means ± SEM of 5 mice per group from 2–3 separate experiments and 5 views per mouse. ***p<0.001 (one way ANOVA with Tukey). (G) TNFα levels determined from explanted cecal supernatants by ELISA. (H) Fecal lipocalin-2 levels determined from fecal pellets on day 5 after *Salmonella* infection. Data are means with SEM from 2–3 experiments. **p<0.01, ***p< 0.001 (Mann-Whitney test).

We previously discovered a genomic island (PI-6) on an extrachromosomal plasmid in AIEC strain NRG857c that confers resistance to human host defense peptides through the action of antimicrobial peptide resistance locus, *arlABC*. ArlA is a Mig-14 family member protein implicated in defensin resistance in *Salmonella*, ArlB is a predicted NAD-dependent epimerase, and ArlC is an OmpT family outer membrane protease that cleaves cationic antimicrobial peptides [33, 34]. In order to test whether the worsened disease outcome following infectious gastroenteritis was directly linked to expansion of AIEC in the gut, we colonized mice with an AIEC ΔPI-6 mutant that is sensitive to killing by defensins, cathelicidins, and by the antimicrobial activity of the monokine MIG/CXCL9 [34], and tested whether this peptide-sensitive mutant could expand in the post-infectious period following gastroenteritis. Consistent with previous experiments, wild type AIEC expanded 2–3 logs in the cecum and ileum following *S*. Typhimurium gastroenteritis and lead to ~50% host mortality. In contrast, the ΔPI-6 mutant showed no expansion following infectious gastroenteritis in either the cecum or the ileum and none of these co-infected mice succumbed to infection (Fig 5A and 5B), providing a direct link between AIEC expansion and disease outcome. To confirm that fulminant gastroenteritis elicited by *S*. Typhimurium was required for maximal AIEC expansion and for AIEC-dependent pathology, we used an avirulent *Salmonella* mutant that invokes less host inflammation due to loss of the type 3 secretion system-1 (T3SS-1) and T3SS-2 [29] (Δ*invA* Δ*ssaR; labeled S*.*tm* ^*Avir*^ *)*. Co-infection with this *Salmonella* mutant blunted the AIEC expansion compared to that seen following wild type *Salmonella* infection in both the cecum (Fig 5C) and ileum (Fig 5D). Histochemical analysis of H&E-stained cecal sections showed that preventing AIEC expansion mitigated the tissue pathology compared to mice in which wild type AIEC blooms were present (Fig 5E and 5F). In mice colonized with AIEC ΔPI-6, crypt architecture and goblet cells were largely intact and there were fewer necrotic cells in the lumen. Also, when *S*.*tm* ^*Avir*^ was used for the secondary infection in AIEC-colonized mice there was a significant reduction in immunopathology (Fig 5F), a blunted TNFα release from explanted cecal tissue (Fig 5G), and a significant reduction in fecal lipocalin-2 output (Fig 5H).

### Infectious colitis prevents convalescence from AIEC colonization

**Fig 6:**
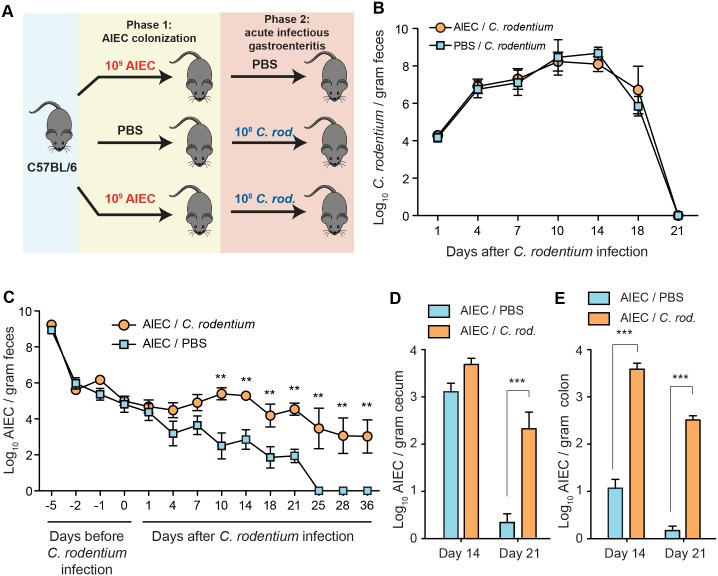
Infectious colitis by *C*. *rodentium* prevents convalescence from AIEC colonization. (A) Infection scheme. C57BL/6 mice colonized with 2 x 10^9^ cfu AIEC NRG857c for 7 days or treated with PBS and then infected with 1 x 10^8^ cfu *C*. *rodentium* or given PBS. (B) Fecal shedding of *C*. *rodentium* was monitored for 21 days. Data are means ± SEM of at least 5 mice per group/time point from 3 independent experiments. (C) Fecal shedding of AIEC after *C*. *rodentium* infection was monitored for 36 days. Data are means ± SEM of at least 5 mice per group/time point from 3 independent experiments. Tissue- associated AIEC was measured in the cecum (D) and colon (E) on day 14 and 21 after *C*. *rodentium* infection. Data are means ± SEM of at least 5 mice per group/time point from 3 independent experiments. **p<0.01 and ***p<0.001 (Mann-Whitney test).

In addition to *Salmonella* gastroenteritis being a risk factor for new onset CD [18], enteropathogenic *E*. *coli* (EPEC) has been associated with both first onset IBD [16, 17, 35] and relapsing disease requiring surgical intervention [23, 24]. The *Citrobacter rodentium* murine model of infectious colitis, resembling the disease course of EPEC in humans, is well established [36]. The disease course in C57BL/6 mice is acute and self-limiting, providing an opportunity to validate our findings on AIEC potentiation in a secondary co-infection model. To test this we colonized groups of C57BL/6 mice with AIEC, which, in contrast to 129e mice that are colonized for the lifetime of the animal, are colonized transiently with AIEC over a 3–4 week timeframe [25]. Infectious colitis was initiated 7 days after AIEC colonization by secondary exposure to *C*. *rodentium* (Fig 6A), and bacterial loads in the feces, cecum and colon were tracked over the next 36 days. Both AIEC-naïve and AIEC-colonized C57BL/6 mice cleared *C*. *rodentium* infection in a reproducible fashion by day 21, indicating that resident AIEC did not alter host immunity towards *C*. *rodentium* (Fig 6B). Similar to what we observed in previous studies, C57BL/6 mice monocolonized with AIEC cleared the bacteria around day 25 (Fig 6C). In contrast, *C*. *rodentium* infection caused an outgrowth of AIEC, which was detectable in the feces beginning ~ 4 days after *C*. *rodentium* infection and reaching significance by day 10 and thereafter until day 36 when the experiment was stopped (Fig 6C). Interestingly, this AIEC expansion persisted into the post-infectious period after *C*. *rodentium* was cleared, and lasted well after monocolonized mice typically clear AIEC, indicating that infectious colitis prevents normal convalescence from AIEC colonization in this host background. *C*. *rodentium* infection initiates in the cecum and progresses to the colon. These sites are the primary targets of host inflammation whereas the small intestine is generally neither colonized nor inflamed by *C*. *rodentium* [37]. We measured the tissue-associated AIEC loads at these sites and found a significant AIEC bloom in the cecum at day 21, and in the colon at both day 14 and 21 after *C*. *rodentium* infection (Fig 6D and 6E).

### Mucosal epithelial restitution is delayed by AIEC following infectious colitis

**Fig 7:**
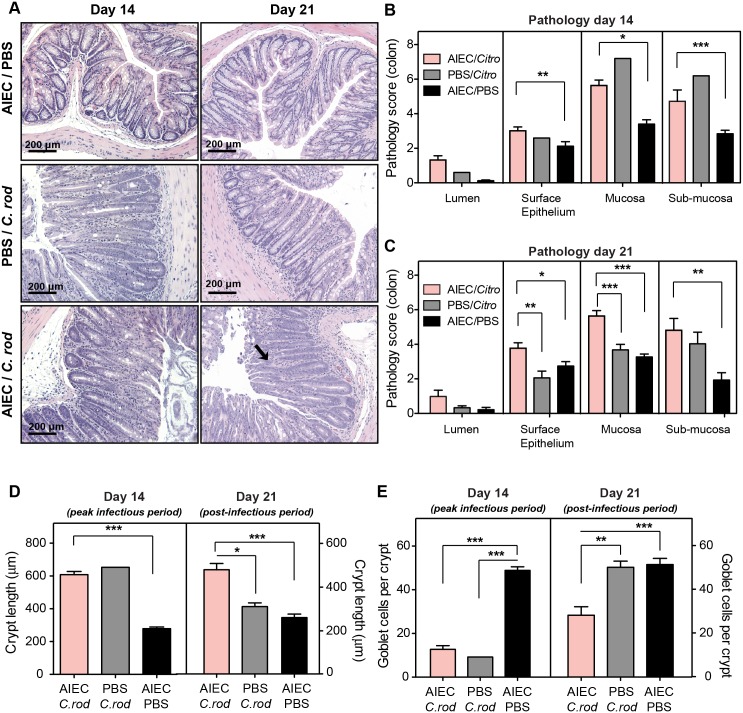
Mucosal epithelial restitution is delayed by AIEC following infectious colitis. (A) H&E staining of colonic sections from C57BL/6 mice infected as indicated, on day 14 and day 21 after *C*. *rodentium* infection. Images are representative of 2 experiments with 5 mice per group per time point. Arrows indicate submucosa edema, desquamation, hyperplasia, inflammatory cellular infiltrates and epithelial sloughing. Original magnification 200x. Quantification of colonic pathology on day 14 (B) and day 21 (C) after *C*. *rodentium* infection. Measurements represent an average of at least 5 views per section and are the means with SEM from 5 mice. Quantification of crypt length (D) and goblet cell numbers (E) on day 14 and day 21 after *C*. *rodentium* infection. Measurements are from at least 5 views per section. Data are expressed as the means ±SEM of 5 mice per group/time point from 2 separate experiments. *p<0.05, **p<0.01, and ***p<0.001 (one way ANOVA with Tukey).

*C*. *rodentium* levels in the colon peak 10 days after infection, followed by the onset of peak inflammation at day 14 when pathology is maximal. Histological analyses of H&E-stained colonic sections showed that by day 14, *C*. *rodentium* had induced colon pathology in both AIEC-colonized and AIEC-naïve hosts (Fig 7A). This early pathology was primarily driven by *C*. *rodentium* because the degree of pathology was similar in both infection groups (Fig 7B). By day 21, the pathogenic features in *C*. *rodentium* monocolonized mice were largely normalized, however significant pathological features persisted on day 21 in AIEC-colonized mice exposed to *C*. *rodentium* despite the fact that *C*. *rodentium* had been cleared by this time point. The surface epithelium and mucosa were the main sites of persistent host damage, and to a lesser extent the sub-mucosa (Fig 7C), indicating that AIEC potentiated tissue damage in the post-infectious period.

*C*. *rodentium* induces transient crypt hyperplasia and goblet cell depletion in the colon that begins to normalize following the peak inflammatory period as the pathogen is cleared by ~ day 21 [36, 38]. On day 14, both infection groups exposed to *C*. *rodentium* had significant crypt hyperplasia (Fig 7D) and goblet cell depletion (Fig 7E) compared to mice singly colonized with AIEC. However on day 21, a time point at which *C*. *rodentium* had been cleared and the crypt length and goblet cell phenotype had normalized in mice singly infected with *C*. *rodentium* (Fig 7D and 7E), mice harbouring AIEC prior to infectious colitis had a significant defect in mucosal reconstitution, displaying persistent crypt hyperplasia and a sustained defect in goblet cell numbers (Fig 7D and 7E). Given the high AIEC expansion in the colon at this time point, these findings indicated that AIEC drives intestinal pathology and delayed mucosal epithelial restitution in the post-infectious period following infectious colitis.

## Discussion

Despite decades of research, a unifying etiologic pathway to CD has been elusive. Genome-wide association studies have now revealed close to 200 IBD susceptibility alleles. However, in aggregate these loci account for only ~30% of CD heritability with a dominant contribution of three common *NOD2* variants [39]. The discordance rate of CD among monozygotic twins also highlights the importance of environmental factors in disease pathogenesis [30]. Thus, while genetic susceptibility remains a key consideration for risk assessment it must be considered among a constellation of other additive risk factors. Recent attention has turned towards a ‘multi-hit’ model of CD pathogenesis that reflects this conceptual framework [19]. In this model the cumulative effects of several factors presents a ‘tipping point’ between homeostasis and protracted intestinal inflammation, a concept that was recently validated in a mouse model involving a genetic TLR1 deficiency [40]. Another risk factor in this multi-hit framework is acute infectious gastroenteritis caused by exposure to relatively common zoonotic pathogens [16–18]. Although such infections tend to be self-limiting, these episodes confer long-term risk of CD well into the post-infectious period, suggesting a sustained imbalance of intestinal homeostasis. Given that healthy subjects can carry AIEC as part of the microbial composition in their gut [41], one possibility is that infectious gastroenteritis elicits the pathogenic character of quiescent AIEC that subsequently exacerbates host damage. Indeed, our report suggests that Crohn’s-associated AIEC exploit the unique host environment imprinted by the action of pathogenic microbes that have been linked to short- and long-term risk of IBD.

AIEC expansion and host damage following *S*. *Typhimurium* gastroenteritis required the resistance of AIEC to host defense peptides. One of the first CD susceptibility genes was *NOD2/CARD15*, in which loss-of-function mutations increase the risk of disease [42, 43]. Early studies reported that loss of NOD2 signaling produced defects in the manufacture of some human defensins by Paneth cells and offered a mechanism to account for the altered microbial composition in CD. However these data have been challenged based on human [44, 45] and better-controlled mouse studies [46] that show antimicrobial peptide production by Paneth cells appears to be independent of *NOD2* status. This is more in keeping with observations that many antimicrobial peptides are instead increased in inflamed Crohn’s lesions [47, 48]. It also implies that bacteria living at the mucosal surface with an active role in Crohn’s pathogenesis would require mechanisms to resist antimicrobial peptides in the inflamed gut, at least in some patients. Indeed, we found that when AIEC were rendered susceptible to host defense peptides they could no longer expand following infectious gastroenteritis and produced moderating effects on disease severity. Previous work found that, among AIEC isolates from IBD patients that were phenotypically resistant to antimicrobial peptides (MIC >8 μg/ml), many of these strains contained at least one resistance gene from the *arlABC* locus [34]. Interestingly, none of the *E*. *coli* isolated from healthy subjects were resistant to host defense peptides, suggesting that the host might imprint a selective pressure for the evolution of such traits. Whether this selective host environment arises as a result of genetics, the endogenous microbiota, or even xenobiotics used in the management of CD remains an open question. The fact that AIEC are genetically diverse [12] suggests that a number of different founder populations are capable of giving rise to this pathobiont type and understanding what shapes this evolutionary trajectory will be an important topic for future studies.

The effect of infectious gastroenteritis on eliciting the pathobiont characteristics of AIEC is consistent with other inflammation models using either chemical colitis or host genetic deficiencies. For example, mouse colon injured by the sodium salt of dextran sulfate produces pan-colitis that is further aggravated by AIEC strain LF82 [49, 50]. A genetic deficiency model using TLR5^-/-^ mice that can develop spontaneous colitis showed that transient colonization by AIEC strain LF82 promoted the development of a colitic microbiota that persisted after AIEC was cleared by the host [51, 52]. At this time, we cannot rule out wholesale changes in the microbiota of our co-infection model in favor of a more proinflammatory composition, however our data suggests this is not the main driver. The expansion of tissue-associated AIEC and its direct correlation with pathological outcomes in our study is consistent with human studies showing that the severity of ileal CD is directly correlated with the tissue-associated *E*. *coli* burden [3–5]. Thus, if global changes in the microbiota are present, it does not alter the disease course in the absence of AIEC expansion.

In summary, exposure to pathogens that trigger acute gastroenteritis creates an environment favorable to colonization by AIEC in otherwise uncontrived hosts. AIEC can be detected in a proportion of healthy individuals, which bears relevance to the finding that infectious gastroenteritis in the general population is a risk factor for IBD. This work provides rationale for the development of novel diagnostic methods that could help identify AIEC-colonized individuals who may be at greater risk following an episode of acute gastroenteritis. It is also possible that infectious gastroenteritis in humans renders them more susceptible to *de novo* acquisition of AIEC through vulnerabilities created by the disruption of the resident microbiota, a loss of so-called colonization resistance. If so, efforts to understand the provenance of AIEC and its transmission mechanisms should be intensified. Finally, the often long prodromal period for CD [53] and the observed latency between recovery from acute gastroenteritis and CD onset may create intervention opportunities to mitigate or neutralize disease risk in a subset of individuals.

## Methods

### Ethics statement

Animal experiments were conducted according to guidelines set by the Canadian Council on Animal Care using protocols approved by the Animal Review Ethics Board at McMaster University under Animal Use Protocol #13-07-20.

### Bacterial strains

AIEC strain NRG857c (serotype O83:H1) was isolated from an ileal tissue biopsy from a CD patient in Charite Hospital (Berlin, Germany) and its genome sequence was determined previously [10, 54]. A mutant that is sensitized to the action of host defense peptides by deletion of the genes *arlABC* (NRG857c ΔPI6) was characterized previously [34]. NRG857c and ΔPI-6 were cultured in Luria-Bertani (LB) broth containing chloramphenicol (34 μg/mL) and ampicillin (100 μg/mL). *Salmonella enterica* serovar Typhimurium strain SL1344 was cultured in LB broth containing 50 μg/ml streptomycin. *Escherichia coli* K-12 strain MG1655 was cultured in LB broth with 50 μg/ml rifampicin. *Citrobacter rodentium* strain DBS100 was cultured in LB broth with no antibiotics. For mouse infections, AIEC was grown for 16–18 h at 37°C with shaking, washed, diluted, and resuspended in phosphate buffered saline (PBS). Prior to infection experiments *S*. *Typhimurium* was resuspended in HEPES buffer (pH 8.0), 0.9% NaCl.

### Animal infections

Eight- to ten-week-old female 129e and C57BL/6 mice were purchased from Charles River Laboratories. Animals were housed in a specific pathogen-free barrier unit under Level 2 conditions at the Central Animal Facility at McMaster University. 129e mice were given 20 mg of streptomycin by orogastric gavage 24 h before infection with 2 x 10^9^ colony forming units (cfu) of AIEC NRG857c. Control groups remained AIEC naïve by gavage with sterile PBS. Two weeks later, AIEC-colonized and control mice were infected with 0.8 x 10^8^ cfu of *S*. *Typhimurium*. In the *C*. *rodentium* model, C57BL/6 mice were challenged with 2 x 10^8^ cfu of *C*. *rodentium* at 1-week post-AIEC infection. Control groups received an equal volume of sterile PBS.

### Bacterial enumeration in tissues

AIEC and the secondary acute pathogen were measured in fecal output. At various time points after secondary infections, tissue-associated bacteria were enumerated in the cecum, colon and ileum. Tissues were harvested into cold PBS at necropsy and flushed with PBS. Samples were homogenized (Retsch), serially diluted in PBS, and plated on LB agar containing either chloramphenicol and ampicillin to select for AIEC NRG857c; rifampicin to select for K-12; Brilliant green (BG) agar with no antibiotics to select for *C*. *rodentium*; or BG containing streptomycin to select *S*. *Typhimurium*. After 24 h of incubation at 37°C, colonies were counted and expressed as cfu per gram of tissue.

### Histopathological evaluation and immunohistochemical (IHC) analysis

At various time points after secondary infection, sections of the cecal tip, distal colon or ileum were collected and fixed in buffered 10% formalin for 72 h, paraffin-embedded, sectioned into 5-μm slices and then stained with haematoxylin and eosin (H&E) by Histology Services (McMaster University, Hamilton, ON). In some experiments tissues were flash frozen in optimal cutting template compound (OCT; Sakura, Fisher) for immunohistochemical analysis. A minimum of 5 views per section were analyzed for each sample and scored according to previously defined criteria summarized in S1 Table [25]. Crypt length measurements and goblet cell quantification was done using ImageJ software on a Leica HC microscope with at least five well-oriented crypts measured per field. Immunohistochemical staining of formalin- and OCT- fixed tissue sections were performed using antibodies against F4/80 (1:600), CD3 (1:1000) and GR1 (1:500) at the Histology Services. Quantification was done on six to eight views per section with at least six formalin-fixed sections per group.

### Cytokine quantification

Tissues removed at necropsy on day 5 following infection with the secondary pathogen were washed with cRPMI (10% fetal bovine serum, 1% L-glutamine and 50 μg/mL gentamicin), cut into pieces, placed in 1 mL of cRPMI and incubated overnight at 37°C, 5% CO_2_. Supernatants were removed after incubation and levels of cytokines and chemokines were determined using the Mouse 32-Plex Discovery Assay by Eve Technologies (Calgary, AB) or Quantikine murine ELISA kits from R&D systems (Minneapolis, MN).

### Intestinal permeability assay

To measure intestinal permeability, mice were gavaged with 150 μl of 80 mg/ml FITC dextran (4kDa) in PBS 4 h prior to sacrifice. Blood was collected by cardiac puncture or tail bleed into 15% v/v acid-citrate- dextrose. Plasma was collected by centrifugation and fluorescence was measured at 530 nm with excitation at 485 nm.

### Nitrite determination

Nitrite concentration in cecal supernatants was determined in a 96-well microplate by adding 50 μl of supernatants to 50 μl of Greiss reagent (Sigma-Aldrich) following incubation for at least 10 min in the dark as described in the manufacturer’s protocol. The absorbance (*A*_550_) was measured and the concentration was determined from a standard curve.

### Lipocalin determination

Fecal pellets were collected, weighed, homogenized in PBS using a Mixer Mill and then centrifuged at 13,000 rpm for 5 min. Following centrifugation, supernatants were removed and levels of lipocalin were determined using a Quantikine murine ELISA kit from R&D systems (Minneapolis, MN).

### Statistical analysis

Mann-Whitney or one-way ANOVA with Tukey or Dunnett post-test was performed using a 95% confidence interval to determine difference among infection groups. Kaplan-Meier survival curves were analyzed with the log rank test. All analyses were performed using Graph Prism 5.0 (GraphPad Software Inc. San Diego, CA). A *P* value of 0.05 or less was considered significant.

## Supporting Information

S1 Table Histopathological scoring for colonic and cecal tissue. (DOCX)
