# Correction: Acute Infectious Gastroenteritis Potentiates a Crohn's Disease Pathobiont to Fuel Ongoing Inflammation in the Post-Infectious Period

**DOI:** 10.1371/journal.ppat.1007032

**Published:** 2018-04-30

**Authors:** Cherrie L. Small, Lydia Xing, Joseph B. McPhee, Hong T. Law, Brian K. Coombes

The authors would like to correct Fig 5. During the preparation of the manuscript, two incorrect H&E images were used in Fig 5E for the AIEC/PBS and AIEC/*S*.*tm* groups. The images depicted in the original version of the Figure represent the correct experimental groups, however the images were not the correct images for the Figure. The conclusions of the manuscript are not changed.

**Fig 5:**
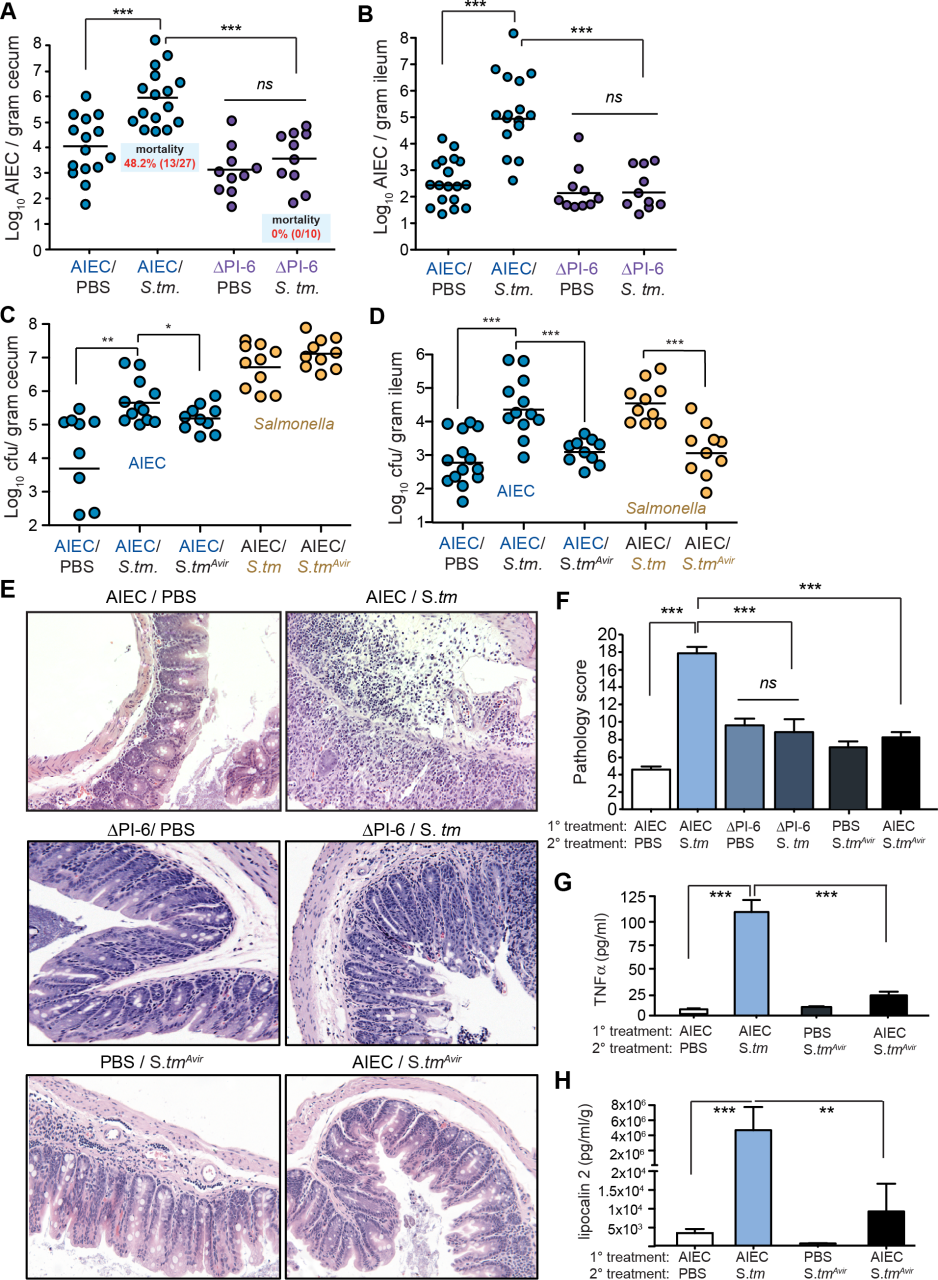
Resistance to host defense peptides is required for AIEC expansion in the inflamed gut and immunopathology. 129e mice were colonized with wild type AIEC or the peptide-sensitive ΔPI-6 mutant and then infected with *Salmonella*. Survival was assessed over 35 days and the tissue burden of AIEC was determined in the cecum (A) and the ileum (B). Each point represents one animal and the data represents 3 independent experiments. ***p< 0.001 (Mann-Whitney test). 129e mice were colonized with AIEC and then infected with either wild type *Salmonella* or a mutant that is less proinflammatory, *S*.*tmavir*. Tissue burden of AIEC (blue circles) and *Salmonella* (yellow circles) was determined in the cecum (C) and the ileum (D). Each point represents one animal and the data represents 2 independent experiments. *p<0.05; **p<0.01; ***p< 0.001 (Mann-Whitney test). (E) H&E-stained tissue samples from cecal tips are shown for the indicated infection groups. Original magnification 200x. (F) Quantification of histopathology from part E. Data are the means ± SEM of 5 mice per group from 2–3 separate experiments and 5 views per mouse. ***p<0.001 (one way ANOVA with Tukey). (G) TNFα levels determined from explanted cecal supernatants by ELISA. (H) Fecal lipocalin-2 levels determined from fecal pellets on day 5 after *Salmonella* infection. Data are means with SEM from 2–3 experiments. **p<0.01, ***p< 0.001 (Mann-Whitney test).
