# Supplementary material for: Acute Infectious Gastroenteritis Potentiates a Crohn's Disease Pathobiont to Fuel Ongoing Inflammation in the Post-Infectious Period
Source: PLoS Pathog. 2016 Oct 6;12(10):e1005907. doi: 10.1371/journal.ppat.1005907 (PMC5053483; doi:10.1371/journal.ppat.1005907)
Supplement: S1 Table — (DOCX) [file ppat.1005907.s001.docx]

**Table S1: Histopathological scoring for colonic and cecal tissue.**

| **LAYER** | **PATHOLOGICAL CHARACTERISTICS** | **SCORE** | **TOTAL** |
| --- | --- | --- | --- |
| **Lumen** | empty | (0) |  |
|  | Sloughed epithelial cells | scant (1)  moderate (2)  dense (3) |  |
|  | PMNs | < 5 PMNs (0)  5-20 PMNs (1)  21+ PMNs (2) |  |
| **Total (lumen)** |  |  | **5** |
| **Surface Epithelium** | Epithelial Integrity | No Pathological Changes (0)  Desquamation (1)  Epithelial Erosion (2)  Ulceration (3) |  |
| **Total (SE)** |  |  | **3** |
| **Mucosa** | Loss of Crypts | Rare, <15% (1)  Moderate, 15-20% (2)  Abundant, >50% (3) |  |
|  | PMN infiltration | < 5 PMNs (0)  5-10 PMNs (1)  21-60 PMNs (2)  >60 PMNs (3) |  |
|  | Crypt hyperplasia | Absent (0)  Present (1) |  |
|  | Crypt distortion | Crypt shortening (1)  Mucosal atrophy (shortening and budding of crypts (2) |  |
|  | Loss of goblet cells | > 28 goblet cells(0)  11-28 goblet cells (1)  1- 10 goblet cells (2)  <1 goblet cell (3) |  |
|  | Granulomatous crypt abscesses | (1) |  |
| **Total (mucosa)** |  |  | **13** |
| **Submucosa** | Mononuclear cell infiltrate | 1 small aggregate (0)  >1 aggregate (1)  large aggregates plus single cells (2) |  |
|  | PMN infiltrate | No extravascular PMNs (0)  Single extravascular PMNs (1)  PMN aggregates (2) |  |
|  | Lymphocytic infiltrates | No lymphocytes (0)  <10 aggregates (1)  >50 aggregates (2) |  |
|  | Edema | Mild (1)  Moderate (2)  Severe (3) |  |
| **Total (SM)** |  |  | **9** |

**Additional Notes:**

**Submucosal edema.** (0) no pathological changes; (1) mild edema (the submucosa is 0.20 mm wide and accounts for 50% of the diameter of the entire intestinal wall [tunica muscularis to epithelium]); (2) moderate edema; the submucosa is 0.21 to 0.45 mm wide and accounts for 50 to 80% of the diameter of the entire intestinal wall; and (3) profound edema (the submucosa is 0.46 mm wide and accounts for 80% of the diameter of the entire intestinal wall). The submucosa widths were determined by quantitative microscopy and represent the averages of 30 evenly spaced radial measurements of the distance between the tunica muscularis and the lamina muscularis mucosae.

**PMN infiltration into the lamina propria.** Polymorphonuclear granulocytes (PMN) in the lamina propria were enumerated in 10 high-power fields (400 magnification; field diameter of 420 mm), and the average number of PMN/high-power field was calculated. The scores were defined as follows: 0-5 PMN/high-power field; 1, 5 to 20 PMN/high-power field; 2, 21 to 60/high-power field; 3, 61 to 100/high-power field; and 4, 100/high-power field. Transmigration of PMN into the intestinal lumen was consistently observed when the number of PMN was more than 60 PMN/high-power field.

**Goblet cells.** The average number of goblet cells per high-power field (magnification, X400) was determined from 10 different regions of the cecal epithelium. Scoring was as follows: 0 - 28 goblet cells/high-power field (magnification, X 400; in the cecum of the normal SPF mice we observed an average of 6.4 crypts/high-power field and the average crypt consisted of 35 to 42 epithelial cells, 25 to 35% of which were differentiated into goblet cells); 1, 11 to 28 goblet cells/high-power field; 2, 1 to 10 goblet cells/high-power field; and 3, less than 1 goblet cell/high-power field.

**Epithelial integrity.** Epithelial integrity was scored as follows: 0, no pathological changes detectable in 10 high-power fields (X400 magnification); 1, epithelial desquamation; 2, erosion of the epithelial surface (gaps of 1 to 10 epithelial cells/lesion); 3, epithelial ulceration (gaps of 10 epithelial cells/lesion; at this stage, there is generally granulation tissue below the epithelium).
